# Mass transfer of acoustic cavitation bubbles in multi-bubble environment^☆^

**DOI:** 10.1016/j.ultsonch.2025.107295

**Published:** 2025-02-27

**Authors:** Kanji D. Hattori, Takuya Yamamoto

**Affiliations:** Department of Chemical Engineering, Graduate School of Engineering, Osaka Metropolitan University, 1-1, Gakuen-cho, Naka-ku, Sakai, Osaka 599-8531, Japan

**Keywords:** Compressive Continuous Species Transfer (C-CST) model, Volume of Fluid (VOF) method, Acoustic cavitation bubble, Mass transfer, Multi-bubble environment, Secondary Bjerknes force

## Abstract

The mass transfer around acoustic cavitation in a multi-bubble environment was numerically studied. To model the multi-bubble environment, several bubbles were placed at the vertices of the polyhedron, and one bubble was placed at the center, and then an ultrasonic pressure wave with a frequency of 20 kHz was imposed along the simulation boundary box. In this study, the mass transfer of chemical species that were initially present only in the bubbles was investigated. The numerical simulation revealed that the mass transfer to the outside bubble was enhanced by the bubble translational motion caused by the secondary Bjerknes force, whereas the mass transfer to the outside bubble increased during the bubble compression period, and the mass transfer to the inside bubble increased during the bubble expansion period. In addition, the mass transfer to the outside bubble was enhanced, particularly for the nonspherical bubble motion during the bubble compression period. The mass transfer to the outside bubble increases with a denser bubble arrangement, which can be explained in terms of the cover ratio, which is defined as the ratio of the projected area of the surrounding bubbles to the central bubble.

## Highlight

• Mass transfer of acoustic bubbles is investigated in a multi-bubble environment.
• It is enhanced due to its translational motion caused by secondary Bjerknes force.
• Non-spherical bubble compression also enhances the mass transfer.
• Mass transfer toward bubble outside increases with the denser bubble arrangement.

## 1 Introduction

When an ultrasound with a large pressure amplitude is applied to water, it generates high-frequency local pressure oscillations, causing acoustic cavitation [1]. When the local pressure is significantly reduced, bubble nuclei are generated, and the bubbles grow significantly during ultrasonic oscillations because of bubble coalescence and *rectified diffusion* [2]. Largely grown bubbles oscillate nonlinearly and are rapidly compressed because of the Rayleigh collapse [3]. At the moment of bubble collapse, the internal temperature and pressure of the bubble temporarily exceed 5000 K and 100 atm., respectively, causing thermal decomposition of water and other molecules in the bubble [4], [5], [6]. The decomposition and formation of reactive radicals in the bubble cause numerous chemical reactions near the gas–liquid interface. Thus, sonochemical reactions proceed. In addition, the nonlinear oscillations of acoustic bubbles cause physical effects such as microstreaming [1], shock waves generation [7], liquid jet formation [8], [9], and bubble clustering [10], [11], [12]. Many applications have been proposed using these sonochemical and physical effects, such as atomization [13], [14], [15], organic chemical decomposition [16], [17], emulsification [18], [19], [20], [21], extraction [22], and nanoparticle fabrication [23], [24]. Among these studies, the interaction between bubble dynamic behavior and sonochemical reactions has been thoroughly investigated [6].

Numerous numerical and experimental studies have been conducted on single bubbles to understand the phenomena occurring during ultrasonic irradiation. These studies are summarized and reviewed in the papers [25], [26], [27] and the book [6]. On the other hand, many acoustic cavitation bubbles are generated simultaneously in a general ultrasonic bath. Under multi-bubble conditions, the phenomena become more complicated than under single-bubble conditions. Therefore, the interaction between bubble motion and chemical reactions is difficult to understand. For example, under multi-bubble conditions, bubble string-like structures called streamer [10], [11], [28], [29] and jellyfish structures [30] are formed. Also, the secondary Bjerknes force causes the nonspherical bubble oscillations and the bubble coalescence [31], [32]. In addition, the ultrasound is shielded by the generation of acoustic cavitation, called acoustic shielding [33], [34], [35], which decreases the sonochemical reaction rate. To evaluate the decrease in the sonochemical reaction rate due to bubble–bubble interaction, Yasui *et al*. [36] modeled the bubble motion and chemical reactions for strongly interacting bubbles and found that the interaction between acoustic bubbles reduces bubble expansion. Later, Shen *et al*. [37] found that the bubble oscillation amplitude was suppressed or enhanced depending on the conditions in a multi-bubble condition. We also found that the bubble oscillation amplitude is enhanced through a three-dimensional multiphase flow simulation [38]. Mettin *et al*. [39] investigated the oscillation patterns of bubbles in clusters numerically and experimentally. However, the interaction between the chemical reactions and bubble dynamics behavior in a cloud is still unclear. In addition to the dynamic behavior of the bubble, the chemical reaction rate is also affected by mass transfer, as discussed below.

Mass transfer around acoustic cavitation has been mainly studied for *rectified diffusion* of dissolved gases [1], [2], [40], [41]. Acoustic cavitation bubbles grow because of *rectified diffusion*, which has recently been investigated, particularly for the damping mechanism of vapor bubbles [42], an improved theoretical model [43], and the effect of surface tension [44]. Mass transfer has also been investigated in hydrodynamics and laser cavitation [45], [46], [47]. Meanwhile, the mass transfer of chemical species generated during nonlinear oscillations and tracers around bubbles has received less attention. Koch *et al*. [48] studied the micromixing caused by the collapse of cavitation bubbles near a wall. Peng *et al.* [49] studied the mass transfer of hydroxyl radicals using a 0-dimensional numerical model. We also investigated the mass transfer of chemical species under single-bubble conditions at different ultrasonic frequencies using a three-dimensional multiphase flow simulation [50], [51]. Although these studies have been conducted, to the best of our knowledge, the mass transfer of acoustic cavitation bubbles in a multi-bubble environment has not been elucidated. It is practically impossible to quantitatively evaluate this experimentally. Therefore, in this study, we investigated the mass transfer of acoustic cavitation bubbles in a multi-bubble environment by three-dimensional multiphase flow simulation coupled with a mass transfer model.

## 2 Numerical analysis

To construct the numerical model, we significantly simplified the acoustic cavitation phenomenon. A cavitation bubble oscillates nonlinearly in an ultrasonic environment, and at the moment of bubble collapse, water molecules in the bubble are thermally decomposed, generating radicals and chemical species such as hydroxyl radicals, hydrogen peroxide, and ozone. In this study, we neglected all chemical reactions and focused on the mass transfer of the chemical species from the cavitation bubble to the liquid phase. In addition, as an initial investigation, we investigated the mass transfer under conditions in which the convective and diffusive mass transfers were balanced. It was assumed that certain chemical species were initially distributed uniformly in the bubble.

In the present study, the simulation was performed with the following assumptions.

• The physical properties, except for the density, were constant, and the flow caused by the surface tension gradient, that is, the Marangoni convection, was negligible because the bubble oscillation velocity was much larger than the flow caused by the Marangoni convection.
• The diffusion coefficient used in this simulation was set to be larger than the actual value to consider the condition in which convective and diffusive mass transfers are balanced.
• Evaporation and condensation are neglected for its simplicity.
• Mass transfer across the gas–liquid interface did not affect the fluid flow and temperature distribution.
• The gas and the liquid are Newtonian fluids.
• These phenomena are thermodynamically in local equilibrium near the gas–liquid interface, and the partition of the chemical species follows Henry’s law.
• The volume change due to mass transfer across the bubble interface was assumed to be negligible.
• Buoyancy and gravity are too small to affect the bubble dynamic behavior because of the small size of the acoustic cavitation bubbles.
• The density variation follows the ideal gas equation of state for the gas and the Tait equation of state for the liquid.

In an actual situation, evaporation and condensation affect the bubble motion and the mass transfer characteristics [45], [46], [47], [52], although the phase change is neglected in this simulation for the sake of simplicity of the numerical model. In addition, we considered that the mass transfer across the bubble interface did not affect the fluid flow motion and temperature distribution because, as discussed by Yasui *et al*. [53], the amounts of chemical species, such as O_3_ and H_2_O_2_, which are the main focus of this study, were small.

In this simulation, bubble motion and mass transfer were investigated during one cycle of ultrasonic waves. As investigated in our previous studies [50], [51], the mass transfer was slightly affected by the artificial initial conditions. Therefore, the simulation was continued for two cycles of ultrasonic waves, and the mass transfer characteristics were evaluated for the second cycle. In contrast, the bubble location changed with time, as will be explained later for the multi-bubble environment. Therefore, we evaluated the mass transfer characteristics of the first ultrasonic cycle.

**Fig. 1:**
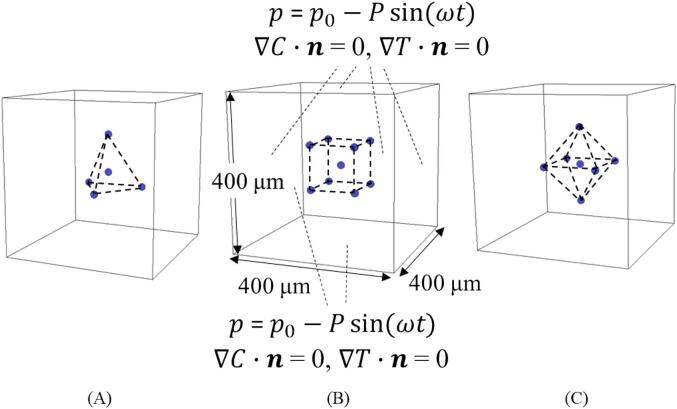
Schematic drawing of calculation domain: A cavitation bubble is surrounded by other bubbles located at the vertices of regular (A) tetrahedron, (B) hexahedron, and (C) octahedron.

The computational domain is illustrated in Fig. 1. The computational domain was a cube with a length of 400 µm. A cavitation bubble was set to be surrounded by several other bubbles located at the vertices of a regular tetrahedron, hexahedron, and octahedron, as in our previous study [38]. Sinusoidal pressure waves were applied to the boundary at a frequency of 20 kHz. The bubble diameter was set to 20 µm. The concentration of the chemical species was non-dimensionalized, and the dimensionless concentration was initially 1 inside the bubbles and 0 outside the bubbles for the initial condition. The pressure amplitudes were varied from 0.50, 0.70, 0.80, 0.85, and 0.90 atm. for the cases in which the distance between the central and the surrounding bubbles was 100 µm or 150 µm. The sinusoidal pressure wave was given by the following equation:

(1) $$p=p_{0}-P\sin\left(\omega t\right)$$

where $p_{0}$ is the atmospheric pressure, P is the pressure amplitude, ω is the angular frequency, and *t* is time. The temperature and concentration gradients normal to the boundary were set to be 0. The numerical simulation was not converged for the case where the distance between the central bubble and the surrounding bubbles was 150 µm, and the bubble arrangement was regular hexahedral. Therefore, numerical results are not presented in this study.

### 2.1 Governing equations

In our numerical simulation, Navier-Stokes, continuity, energy balance, and species mass balance equations were used as the governing equations.

(2) $$\frac{\partial (\rho u)}{\partial t}+\nabla ∙\left(\rho uu\right)=-\nabla p+\nabla ∙\left(\mu \left(\nabla u+\nabla u^{T}-\frac{2}{3}\left(\nabla ∙u\right)I\right)\right)+F_{\sigma }$$

(3) $$\frac{\partial \rho }{\partial t}+\nabla ∙\left(\rho u\right)=0$$

(4) $$\frac{\partial \left(\rho c_{p}T\right)}{\partial t}+\nabla ∙\left(\rho uc_{p}T\right)=\nabla ∙\left(k\nabla T\right)-\left(\nabla ∙\left(pu\right)+\nabla ∙\left(\rho Ku\right)+\frac{\partial \left(\rho K\right)}{\partial t}\right)$$

(5) $$\frac{\partial C}{\partial t}+\nabla ∙\left(Cu\right)=D\nabla^{2}C$$

where ρ is the density, u is the fluid velocity, t is the time, p is the pressure, μ is the viscosity, ***I*** is the unit tensor, $F_{\sigma }$ is the surface tension force, T is the temperature, k is the thermal conductivity, $c_{p}$ is the specific heat capacity, C is the dimensionless concentration of chemical species, D is the diffusion coefficient, and K is the kinematic energy defined as ${\left|u\right|}^{2}/2$.

### 2.2 Multiphase flow model

In this study, an algebraic Volume of Fluid (VOF) method [54] was used. Therefore, the gas–liquid interface was constructed by solving the following conservation equation for the liquid volume fraction:

(6) $$\frac{\partial \alpha }{\partial t}+\nabla ∙\left(u\alpha \right)+\nabla ∙\left(\alpha \left(1-\alpha \right)u_{r}\right)=\alpha \left(1-\alpha \right)\left(\frac{\psi_{g}}{\rho_{g}}-\frac{\psi }{\rho }\right)\frac{\mathrm{Dp}}{\mathrm{Dt}}+\alpha \nabla \cdot u$$

where $\rho_{g}$ is the gas density, ψ is the compressibility, and $u_{r}$ is the relative velocity between the gas and the liquid phase. The volume fraction of liquid α is defined as follows:

(7) $$\alpha =\left\{\begin{array}{c} 1\\ 0<\alpha <1\\ 0 \end{array}\right)\begin{array}{c} Liquid\\ Interface\\ Gas \end{array}$$

In Eq. (6), the third term on the left-hand side (LHS) is modeled as a compressive term. Finally, the relative velocity, $u_{r}$ is finally modeled as the compressive mass flux, *ϕ*_r_. In the finite volume method, the third term on the LHS is discretized as follows:

(8) $$\int_{\Omega }\nabla ∙\left(\alpha \left(1-\alpha \right)u_{r}\right)d\Omega =\sum_{f}\alpha \left(1-\alpha \right)u_{r}\cdot S_{f}=\sum_{f}\alpha \left(1-\alpha \right){\left(\phi_{r}\right)}_{f}$$

where Ω is the cell volume and ***S***_f_ is the area vector of the grid cell face. The compressive flux $\phi_{r}$ is given by

(9) $${\left(\phi_{r}\right)}_{f}=\left(C_{\alpha }\frac{\left|\phi_{f}\right|}{\left|S_{f}\right|}\right)\hat{n_{f}}$$

where $C_{\alpha }$ is the compression parameter and $\hat{n_{f}}$ is the surface normal flux to the gas–liquid interface. The value of $C_{\alpha }$ determines the compressive strength and significantly affects the interfacial capturing accuracy. In the present study, the value of $C_{\alpha }$ is set to 1.0. $\hat{n_{f}}$ is given by the following equation:

(10) $$\hat{n_{f}}=n_{f}∙S_{f}=\left(\frac{{\left(\nabla \tilde{\alpha }\right)}_{f}}{\left|{\left(\nabla \tilde{\alpha }\right)}_{f}+\delta_{s}\right|}\right)∙S_{f}$$

where $n_{f}$ is a vector normal to the gas–liquid interface and $\delta_{s}$ is a stabilization parameter. To improve the calculation accuracy of $n_{f}$, a smoothed volume fraction $\tilde{\alpha }$ is used instead of α[55], [56]. This is because the calculation of $n_{f}$ needs to be accurate in order for compression in a particular direction. $\tilde{\alpha }$ is given by

(11) $$\tilde{\alpha }=\frac{\sum_{f}\alpha_{f}S_{f}}{\sum_{f}S_{f}}$$

In the present study, the number of filters was set to three because threefold filtering greatly reduces the spurious current [57].

To use the multiphase flow model described above, it is necessary to introduce an additional surface tension term into the Navier-Stokes equation. The surface tension term is modeled using the Continuum Surface Force (CSF) model [58] and is given by

(12) $$F_{\sigma }=\sigma k\nabla \alpha$$

where σ is the surface tension, and k is the interface curvature, which is calculated as follows:

(13) $$k=-\int_{\Omega }\nabla ∙nd\Omega =-\int_{S}ndS=-\sum_{f}\hat{n_{f}}$$

### 2.3 Compressive continuous species transfer (C-CST) model

In the present study, the multiphase flow was modeled using the single-fluid model described in Section 2.2. To simulate the mass transfer across the gas–liquid interface with the gas–liquid partition, the compressive continuous species transfer (C-CST) model [59] was used. In this model, the following species balance equation is solved:

(14) $$\frac{\partial C}{\partial t}+\nabla ∙\left(uC\right)=-\nabla ∙\left(\frac{\left(1-He\right)C}{\alpha +He\left(1-\alpha \right)}\alpha \left(1-\alpha \right)u_{r}\right)+\nabla ∙\left(D\nabla C-D\frac{1-He}{\alpha +He\left(1-\alpha \right)}C\nabla \alpha \right)$$

where C is the species concentration, He is Henry’s constant, and *D* is the diffusion coefficient, which is modeled using the equilibrium-based mean formulation [60].

### 2.4 Equation of state (EOS)

In this numerical simulation, the density variation was modeled using the following equation of state to model the compressible bubble motion. In the liquid phase, the following the Tait equation of state was adopted:

(15) $$p=\left(p_{0}+B\right){\left(\frac{\rho }{\rho_{0}}\right)}^{\gamma }-B$$

where $p_{0}$ is the atmosphere pressure, $\rho_{0}$ is the equilibrium density, γ is the dimensionless parameter, and B is the model constant. The values of the parameters used in the Tait equation of state are listed in Table 1.

**Table 1:** Parameters in Tait equation for liquid phase.

$p_{0}\left[Pa\right]$	$\rho_{0}\left[kg/m^{3}\right]$	γ[-]	B[Pa]
1.00 × 10^5^	1.00 × 10^3^	7.15	3.05 × 10^8^

In the gas phase, the following the ideal gas equation of state was adopted:

(16) p=ρRT

where R is the gas constant.

### 2.5 Other numerical methods

The physical properties used in this study are listed in Table 2. In this simulation, the physical properties are air and water for the gas and liquid phases, respectively, and the diffusion coefficient of the chemical species was set such that the Schmidt number (*Sc*) becomes 1 in both the gas and liquid phases when the bubbles are stationary (***u*** = 0), to focus on the condition in which the convective and diffusive mass transfers are balanced. Therefore, the physical properties used in the previous studies [53], [61], [62], [63] were different from those used in this study. The temperature and pressure in the computational domain were initially set to 300 K and 1 atm., respectively.

**Table 2:** Physical properties used in this simulation.

Value	Liquid	Gas
Diffusion coefficient $D[m^{2}/s]$	1.00 × 10^-6^	1.00 × 10^-5^
Viscosity μ[Pa∙s]	1.00 × 10^-3^	1.84 × 10^-5^
Prandtl number Pr[-]	7.00	7.00 × 10^-1^
Heat capacity $c_{p}\left[J/\left(kg∙K\right)\right]$	4.20 × 10^3^	1.01 × 10^3^
Surface tension σ[N/m]	7.25 × 10^-2^	−
Henry’s constant He[-]	3.00 × 10^1^	−

A nonuniform structured grid was used in this simulation. Except for the convective term, the spatial derivatives in the governing equations were discretized using a linear interpolation scheme. For the convective terms, the second term on the left-hand side (LHS) of Eqs. (2) and (4) were discretized using the linear upwind scheme, the third term on the LHS of Eq. (6) and the third term on the right-hand side (RHS) of Eq. (14) were discretized using a linear interpolation scheme, the second term on the LHS of Eqs. (6) and (14), and the first term on the RHS of Eq. (14) were discretized using the van Leer scheme, respectively. The time derivatives were discretized using a the second-order backward scheme, except for that in Eq. (6), which was discretized using the implicit Euler scheme. The basic grid resolution is 200 × 200 × 200, and the grid cell length is 2 µm. The numerical grid was refined near the region where the cavitation bubbles were present. Yamamoto has studied the numerical grid dependency on the mass transfer across the cavitation bubble interface and has shown that the grid size of 0.50 µm is sufficiently fine to evaluate the numerical results [50]. Similar to our previous study, the size of the refined grid cell was set to 0.50 μm. Consequently, the total number of numerical grid cells was approximately 20,000,000–30,000,000, depending on the bubble arrangement.

The accuracy of the numerical model used in this study was verified by Yamamoto [50]; thus, the accuracy of our numerical models is sufficiently high to quantitatively evaluate the mass transfer across the gas–liquid interface. Therefore, the further verification is not addressed again in this paper.

### 2.6 Calculation of quantitative values in a post-process of numerical simulation

The methods for calculating the quantitative values are explained below. The bubble shape becomes nonspherical at a high pressure amplitude; therefore, the equivalent radius was used to quantify the bubble size. The equivalent radius was calculated using the following equation:

(17) $$R_{e}={\left(\frac{3\int_{\Omega }\left(1-\alpha \right)d\Omega }{4\pi }\right)}^{\frac{1}{3}}$$

The average bubble velocity of a surrounding bubble was calculated by the following equation.

(18) $$U=\left|\frac{\int_{\Omega }\left(1-\alpha \right)ud\Omega }{\int_{\Omega }\left(1-\alpha \right)d\Omega }\right|$$

The total amount of chemical species in the bubble was calculated using the following equation [50], [51], [64]:

(19) $$C_{int}=\int_{\Omega }\left(1-\alpha \right)C_{g}d\Omega =\int_{\Omega }\frac{\left(1-\alpha \right)CHe}{\alpha +\left(1-\alpha \right)He}d\Omega$$

The Reynolds number, *Re*, and Sherwood number, *Sh* were defined as follows:

(20) Re=2ReUνl

(21) $$\mathrm{Sh}=\frac{k\left(C_{l,i}-C_{l,\infty }\right)}{-D_{l}\frac{\left(C_{l,\infty }-C_{l,i}\right)}{2R_{e}}}=\frac{2kR_{e}}{D_{l}}$$

where $\nu_{l}$ is the kinematic viscosity in the liquid phase, $C_{l,i}$ and $C_{l,\infty }$ are the concentrations of the chemical species in the liquid phase at the bubble interface and at infinity, respectively; and k is the mass transfer coefficient, which was calculated using the following equation:

(22) $$k=\frac{N}{C_{l,i}-C_{l,\infty }}$$

According to Henry’s law,

(23) $$C_{l,i}=\frac{C_{g,ave}}{\mathrm{He}}$$

where $C_{g,ave}$ is the average concentration of the chemical species in the bubble and was calculated using the following equation:

(24) $$C_{g,ave}=\frac{C_{int}}{\int_{\Omega }\left(1-\alpha \right)d\Omega }=\frac{\int_{\Omega }\frac{\left(1-\alpha \right)CHe}{\alpha +\left(1-\alpha \right)He}d\Omega }{\int_{\Omega }\left(1-\alpha \right)d\Omega }$$

N is the mass flux, which was calculated using the following equation:

(25) $$N=\frac{-\frac{dC_{int}}{dt}}{A_{g}}$$

where, $A_{g}$ is the interface area of the cavitation bubble, calculated using the following equation:

(26) $$A_{g}=\int_{\Omega }\left|\nabla \left(1-\alpha \right)\right|d\Omega$$

## 3 Numerical results

For all arrangements of the surrounding bubbles, the mass transfer around the bubble was similar at all pressure amplitudes. Therefore, in the next subsection, we present numerical results for a specific arrangement of surrounding bubbles as an example.

### 3.1 Dynamic behavior of acoustic cavitation bubbles in a multi-bubble environment

**Fig. 2:**
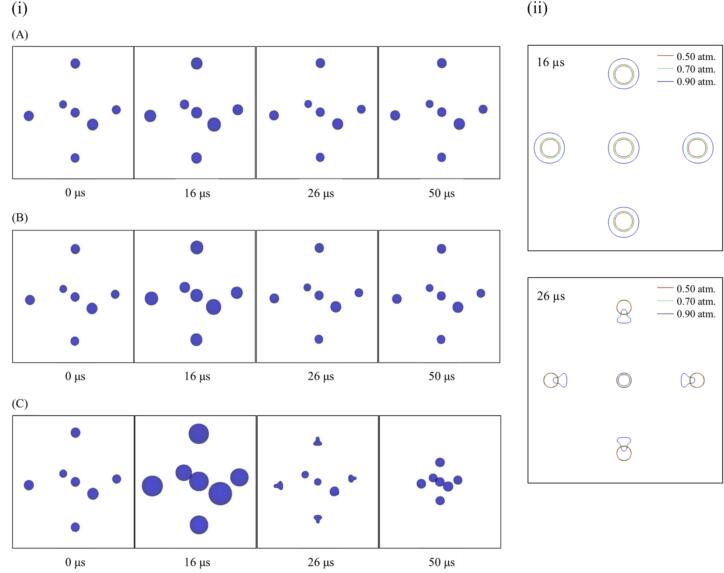
Dynamic bubbles behavior at different pressure amplitudes when the distance between the central bubble and the surrounding bubbles is 100 µm and the bubble arrangement is a regular octahedron. The bubble shapes are drawn (i) in the three-dimensional space and (ii) in the cross-sectional plane. The pressure amplitudes are (A) 0.50 atm, (B) 0.70 atm, and (C) 0.90 atm.

First, we discuss the dynamic behavior of acoustic cavitation bubbles in a multi-bubble environment. Fig. 2 shows the dynamic bubble behavior at different pressure amplitudes when the distance between the central bubble and the surrounding bubbles is 100 µm and the bubble arrangement is the regular octahedron. In this figure, the time at 16 µs and 26 µs correspond to the bubble expansion and compression periods, respectively. As can be seen from Fig. 2, at low-pressure amplitudes, 0.50 and 0.70 atm., the oscillation amplitude of the bubbles is small, and the position of the bubbles is not changed. At a high-pressure amplitude of 0.90 atm., the oscillation amplitude of the bubbles is large, and the surrounding bubbles become nonspherical during the compression period and move toward the central bubble. This movement was caused by the secondary Bjerknes force, which is an attractive force between the bubbles under ultrasound. At a high-pressure amplitude, the bubble oscillations become more intense, attracting more liquid during the compression period and creating a large surrounding liquid flow, causing the bubble to move to the central bubble. In addition, as described above, an asymmetric flow is created around the surrounding bubbles, creating a liquid jet toward the central bubble, and the surrounding bubbles become nonspherical during their oscillations.

**Fig. 3:**
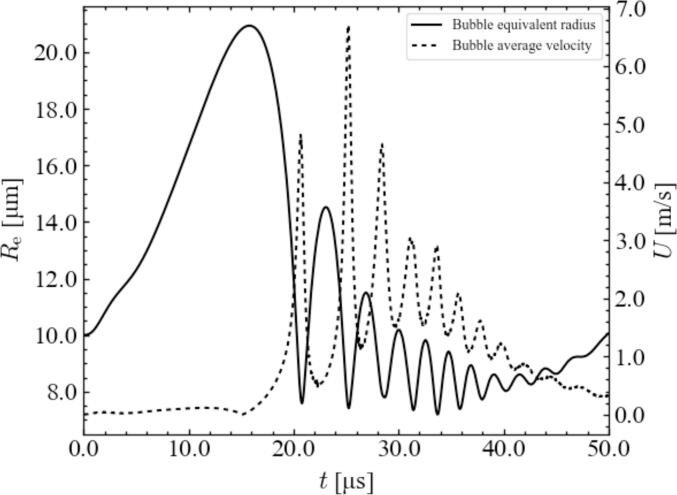
Time variation of bubble equivalent radius and bubble average velocity of a surrounding bubble when the distance between the central bubble and the surrounding bubbles is 100 µm and the bubble arrangement is a regular tetrahedron. The pressure amplitude is 0.90 atm.

To quantitatively evaluate the dynamic behavior of the surrounding bubbles at high pressures, the time variations in the bubble radius and bubble motion velocity were studied. Fig. 3 shows the time variation of the bubble equivalent radius and the bubble average velocity of a surrounding bubble when the distance between the central bubble and the surrounding bubble is 100 µm and the bubble arrangement is a regular tetrahedron at a pressure amplitude of 0.90 atm. As shown in Fig. 3, the average bubble velocity increases as the equivalent radius decreases. The surrounding bubbles move rapidly toward the central bubble during compression. Conversely, the average bubble velocity decreases as the equivalent radius increases. The velocity of the surrounding bubbles decreases during the expansion period. These results can be explained by the amount of liquid around the surrounding bubbles, which is pulled by the bubble oscillations, and the secondary Bjerknes force between the central and surrounding bubbles.

### 3.2 Mass transfer across the interface of acoustic cavitation bubbles in a multi-bubble environment

**Fig. 4:**
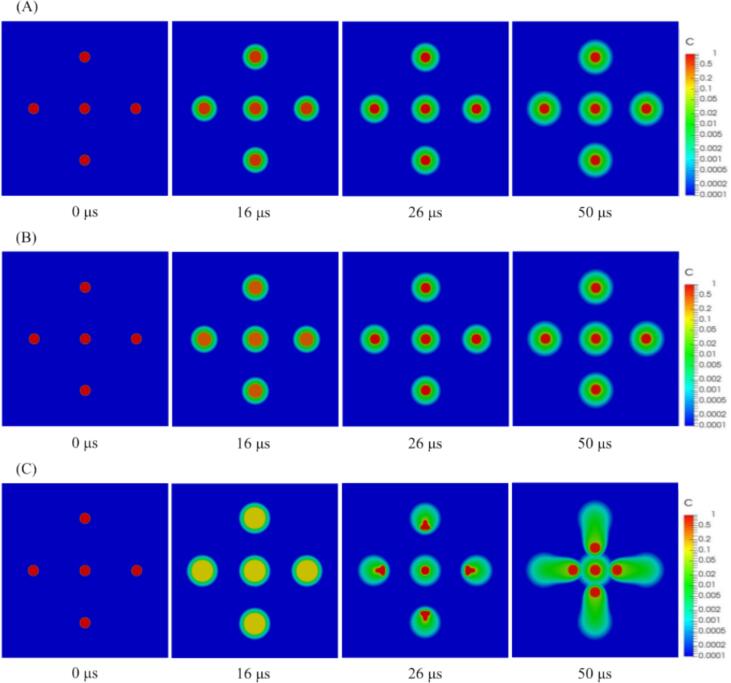
Concentration distribution on the cross-sectional plane at different pressure amplitudes when the distance between the central bubble and surrounding bubbles is 100 µm and the bubble arrangement is a regular octahedron. The pressure amplitudes are (A) 0.50 atm, (B) 0.70 atm, and (C) 0.90 atm.

Fig. 4 shows the time variation of the concentration distribution on the cross-sectional plane at different pressure amplitudes when the distance between the central bubble and the surrounding bubbles is 100 µm and a regular octahedral bubble arrangement. As can be seen from Fig. 4, at the low-pressure amplitudes, 0.50 and 0.70 atm., the location of the bubble center is unchanged, and the chemical species remain in the bubbles, while at a high pressure amplitude, 0.90 atm., the mass transfer to the bubble outside is enhanced along with the bubble movement. As discussed in the previous section, at a high pressure amplitude, the secondary Bjerknes force becomes significantly large, and a liquid jet is generated toward the central bubble, causing the surrounding bubbles to move toward the central bubble with rapid compression. Because of this movement, the bubble surface is renewed, and the low concentration zone in the liquid phase contacts with the bubble surface. In addition, at the moment of compression, the concentration of the chemical species in the bubble increases. To satisfy Henry’s law along the bubble interface, the chemical species are transferred to the outside of the bubbles via translational motion, particularly during the compression period.

**Fig. 5:**
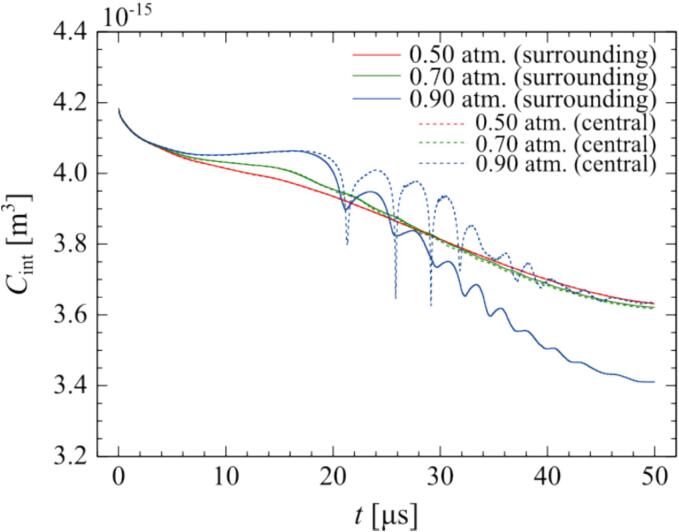
Time variation of total amount of chemical species in the central bubble and one of the surrounding bubbles when the distance between the central bubble and the surrounding bubbles is 100 µm, and the bubble arrangement is a regular octahedron. The pressure amplitudes are 0.50 atm, 0.70 atm, and 0.90 atm.

**Fig. 6:**
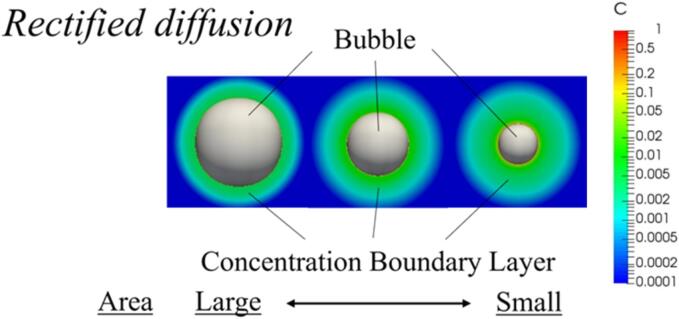
Concentration distribution around a bubble to understand the *rectified diffusion*, *area effect* and *shell effect*.

These results were evaluated quantitatively. Fig. 5 shows the time variation of the total amount of chemical species in the central bubble and in one of the surrounding bubbles at different pressure amplitudes. In this case, the distance between the central bubble and the surrounding bubbles is 100 µm, and the bubble arrangement is a regular octahedron. The total amount of chemical species decreases with time for all pressure amplitudes. Initially, the amount is high inside the bubble and zero outside the bubbles. Therefore, the chemical species is transferred to the liquid phase, causing the amount of chemical species in the bubbles to decrease. At a higher pressure amplitude of 0.90 atm., the amount of chemical species increases or decreases with time, depending on the bubble expansion and compression. Particularly, during the expansion period, the amount of chemical species increases. For the surrounding bubble, the final total amount of chemical species in the bubble in one ultrasonic cycle at the pressure amplitude of 0.90 atm decreases significantly compared to that of the central bubble. This result indicates that the mass transfer to the outside of the bubble is enhanced by the large translational motion caused by the secondary Bjerknes force. For the central bubble, the final total amount of chemical species in the bubble in the ultrasonic cycle increases slightly with increasing pressure amplitude. This result can be partly explained by the *area effect* and the *shell effect* [1], [2]. The *area effect* is caused by the phenomenon in which the mass transfer rate is proportional to the area of the bubble interface. Mass transfer to the inside of the bubble increases during the expansion period, because the area of the bubble interface increases. *Shell effect* is caused by a phenomenon in which the mass transfer rate is proportional to the concentration gradient at the bubble interface in the liquid phase, as shown in Fig. 6. The mass transfer to the inside of the bubble is enhanced during the expansion period, because the concentration boundary layer becomes thinner, and the concentration gradient at the bubble interface becomes large. As the pressure amplitude increases, the bubble oscillation becomes nonlinear, and the *area effect* and *shell effect* become remarkable [50]. Therefore, in the central bubble, a larger amount of chemical species remains in the bubble as the pressure amplitude increases.

**Fig. 7:**
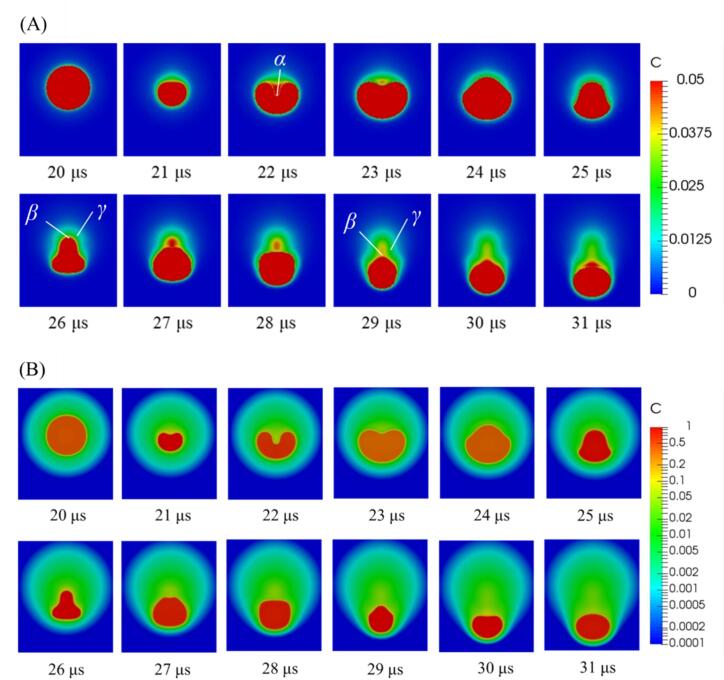
Time variation of concentration distribution near the surrounding bubble on the cross-sectional plane when the distance between the central bubble and the surrounding bubbles is 100 µm, and the bubble arrangement is a regular octahedron. The pressure amplitude is 0.90 atm. The color contour is drawn in (A) normal scale and (B) logarithmic scale.

Fig. 7 shows the time variation of the concentration distribution around the surrounding bubble on the cross-sectional plane at a pressure amplitude of 0.90 atm. when the distance between the central bubble and the surrounding bubbles is 100 µm, and the bubble arrangement is a regular octahedron. The color contour is drawn on (A) a normal scale and (B) a logarithmic scale. The time interval between each snapshot is 1 µ s, with the first snapshot corresponding to 20 µs, just before the liquid jet generation. The bubble becomes asymmetric and the concave interface forms due to asymmetric compression for 20 – 23 µs. After this concave bubble deformation (*t* = 22 – 23 µs), the surface tension and the higher pressure inside the bubble cause the bubble to rebound in the opposite direction. The bubble deformation initiation area, as indicated by *α* in Fig. 7 (A), becomes convex at *t* = 26 µs, and then this bubble oscillation repeats. The time evolution of the concentration distribution on the normal scale (Fig. 7 (A)) shows that the chemical species are transferred to the outside of the bubbles when the convex part of the bubble returns to a spherical shape (Fig. 7 (A) 25 – 30 μs). Thus, the chemical species are transferred to the outside of the bubbles. The concentration around the bubble near the convex part as indicated by *β* in Fig. 7 (A) during the compression period (*t* = 26, 29 μs) is approximately 0.0375 at the bubble interface, while the concentration near the bubble interface (*γ* in Fig. 7 (A)) is less than 0.025, indicating that the mass flux in the liquid phase is to the liquid bulk zone. The concentration distribution on a logarithmic scale, as shown in Fig. 7 (B), shows that the concentration in the bubble increases during the compression period. When the concentration in the bubble increases due to bubble compression, chemical species are transferred to the outside of the bubbles according to Henry’s law. This suggests that a larger mass transfer to the outside of the bubble occurs near the convex part during the compression period.

**Fig. 8:**
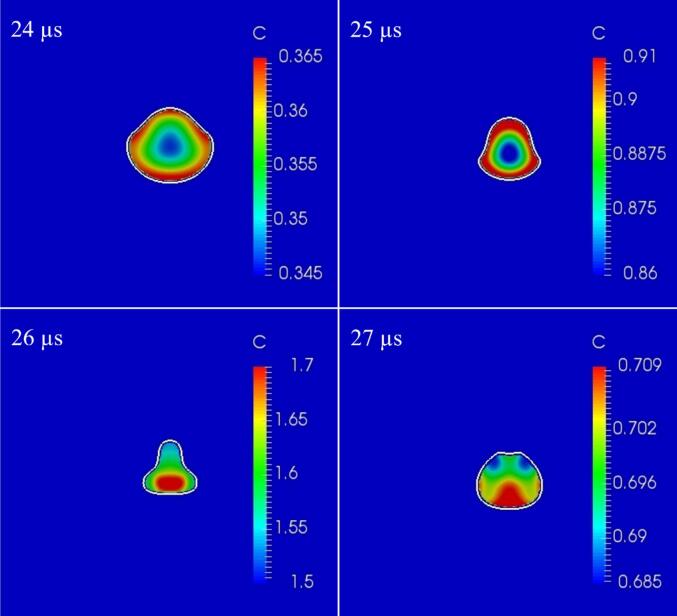
Time variation of concentration distribution in one of the surrounding bubbles during the compression period on the cross-sectional plane when the distance between the central bubble and the surrounding bubbles is 100 µm and the bubble arrangement is a regular octahedron. The pressure amplitude is 0.90 atm. The white line indicates the gas–liquid interface location.

Fig. 8 shows the time variation of the concentration distribution on the cross-sectional plane near one of the surrounding bubbles during the compression period (*t* = 24 – 27 µs). In this case, the pressure amplitude is 0.90 atm., the distance between the central bubble and the surrounding bubbles is 100 µm, and the bubble arrangement is regular octahedral. The concentration in the bubble is higher near the periphery of the gas–liquid interface at 24 – 25 µs, while it is higher at the center of the bubble at 26 – 27 µs. The concentration along the periphery increases during the bubble expansion period because of the *hysteresis effect* [51]. During the bubble expansion period, mass transfer occurs from the outside to the inside of the bubble. In addition, the bubble oscillation period is very short, which decreased the diffusive mass transfer in the bubble. Therefore, the concentration at the periphery is high. This concentration distribution increases the mass transfer during the compression period (25 – 27 µs). The higher concentration along the gas–liquid interface becomes a larger driving force for mass transfer to the outside of the bubble to satisfy the Henry’s law. During the compression period, a large amount of chemical species is transferred into the liquid phase, especially at the location of *β* in Fig. 7 (A). As a result, the concentration inside the bubble decreases near the gas–liquid interface especially near the convex part at 26 µs.

**Fig. 9:**
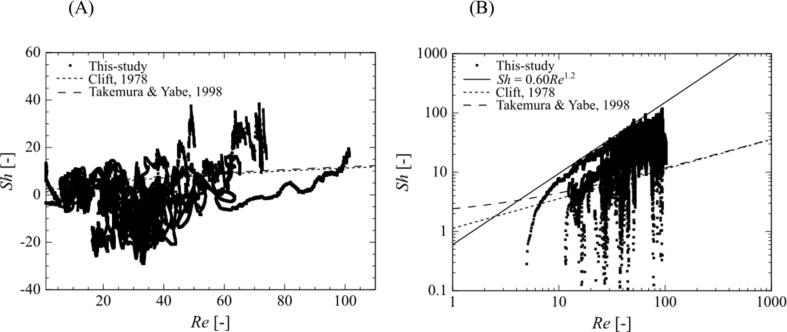
Relationship between the instantaneous Sherwood and Reynolds numbers of a surrounding bubble during the expansion (A) and the compression (B) periods when the distance between the central bubble and the surrounding bubbles is 100 µm, and the bubble arrangement is regular octahedral. The pressure amplitude is 0.90 atm.

The relationship between the bubble motion and mass transfer to the outside bubble was quantitatively evaluated for the surrounding bubble at a high pressure amplitude of 0.90 atm. by using dimensionless numbers. In the present study, the Reynolds number, *Re*, and Sherwood number, *Sh*, were used to evaluate the relationship between the bubble motion and the mass transfer to the outside of the bubble. As defined in Eq. (21), the *Sh* number becomes positive when the mass transfer is to the outside of the bubble and negative when it is to the inside. Fig. 9 shows the relationship between the *Re* and *Sh* numbers calculated using Eqs. (20) and (21) for the expansion (A) and the compression (B) periods, respectively. The numerical results shown in Fig. 9 are plotted for each simulation time increment. The two dashed lines indicate the analytical solution [65] and empirical correlation equation [66] for the mass transfer from spherical bubbles with translational motion. During the expansion period, the most instantaneous *Sh* number is below the analytical and empirical values (Fig. 9 (A)), whereas during the compression period, the most instantaneous *Sh* number exceeds the analytical and empirical values (Fig. 9 (B)), although there is a large variation. Additionally, during the compression period, the maximum *Sh* number for each *Re* number is proportional to the 1.2 power of the *Re* number. These results suggest that the *Sh* number becomes larger than the analytical and empirical values because of bubble compression and nonspherical deformation, as shown in Fig. 8. These results also explain quantitatively why the mass transfer to the outside of the bubble is enhanced by the translational motion of the bubble. In other words, the mass transfer during translational motion can be roughly estimated using these empirical or analytical correlation equations with slight deviations. For example, the Sherwood number is proportional to the square root of the Péclet number, which is the product of the Reynolds and Schmidt numbers. In this study, we used a large diffusion coefficient to investigate the mass transfer under the conditions where the convective and diffusive mass transfers are balanced. When the diffusion coefficient is much smaller, as in the actual diffusion coefficient, the Sherwood number increases, causing a larger mass transfer toward the outside of the bubble.

### 3.3 Scaling law for the mass transfer around acoustic cavitation bubbles in a multi-bubble environment

For all bubble arrangements at a 0.90-atm. pressure amplitude, the mass transfer to the outside of the bubble is enhanced by the movement of the surrounding bubbles toward the central bubble as it is compressed. However, the amount of chemical species released from the bubbles depends on the bubble arrangement and the distance between the bubbles. In a multi-bubble environment, the central bubble is in the shadow of the surrounding bubbles with respect to the sound wave, which causes a delay in sound wave propagation and affects the phase shift and bubble oscillations, as shown in Fig. 11, Fig. 12, Fig. 13, Fig. 14 of our previous study [38]. In addition, the bubble motion is affected by the secondary Bjerknes force. In our previous study, we quantified the changes in bubble motion using the ratio of the shadow of the surrounding bubbles, namely the cover ratio [38], which is the ratio of the projected area of the surrounding bubbles to the central bubble. In this study, we adopted this cover ratio to quantify mass transfer. Here, the cover ratio is defined as:

(27) $$r_{cov}=\frac{\int_{n}S_{cross}dS}{S_{S}}=\frac{n\left(\pi R^{2}\right)}{4\pi X^{2}}=\frac{nR^{2}}{4X^{2}}$$

where $S_{cross}$ is the cross-sectional area of the surrounding bubble, $S_{S}$ is the surface area of the sphere passing through the center point of all surrounding bubbles, n is the number of surrounding bubbles, and X is the distance between the central bubble and the surrounding bubbles. Based on the definitions in Eq. (27), the cover ratio increases with an increase in the volume ratio of the bubbles.

**Fig. 10:**
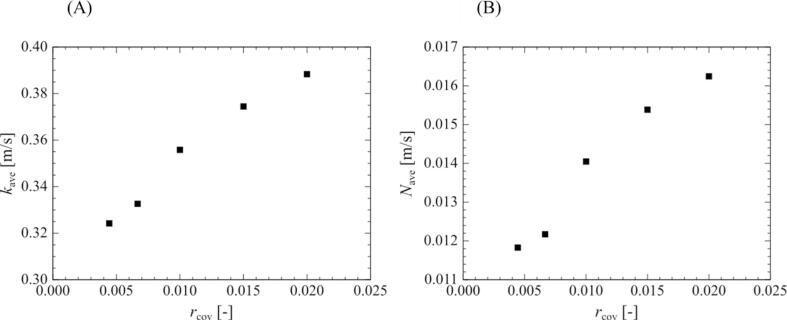
Relationship between the cover ratio and the period-averaged physical quantities related to the mass transfer across the gas–liquid interface of the surrounding bubble. The pressure amplitude is 0.90 atm. The physical quantities are (A) mass transfer coefficient and (B) mass flux.

**Fig. 11:**
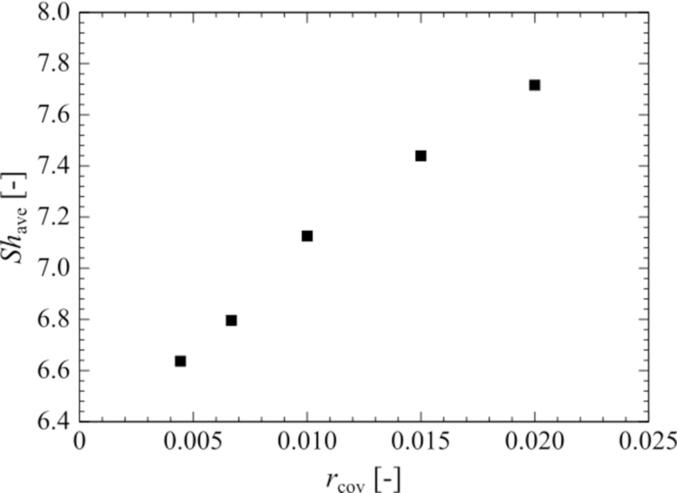
Relationship between the cover ratio and the period-averaged Sherwood number of the surrounding bubble. The pressure amplitude is 0.90 atm.

**Fig. 12:**
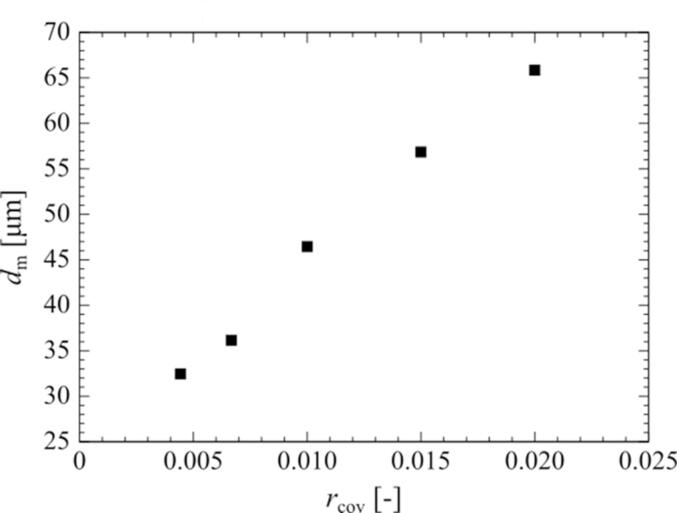
Relationship between the cover ratio and the movement distance of the surrounding bubble in one ultrasonic cycle. The pressure amplitude is 0.90 atm.

**Fig. 13:**
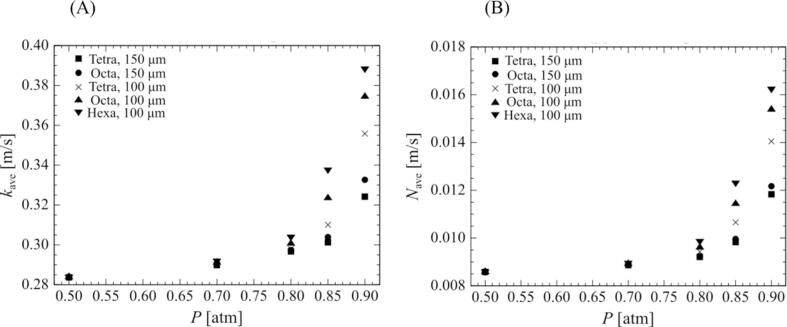
Relationship between the pressure amplitudes and the time-averaged physical quantities related to mass transfer across the gas–liquid interface of the surrounding bubble. The physical quantities are (A) mass transfer coefficient and (B) mass flux.

**Fig. 14:**
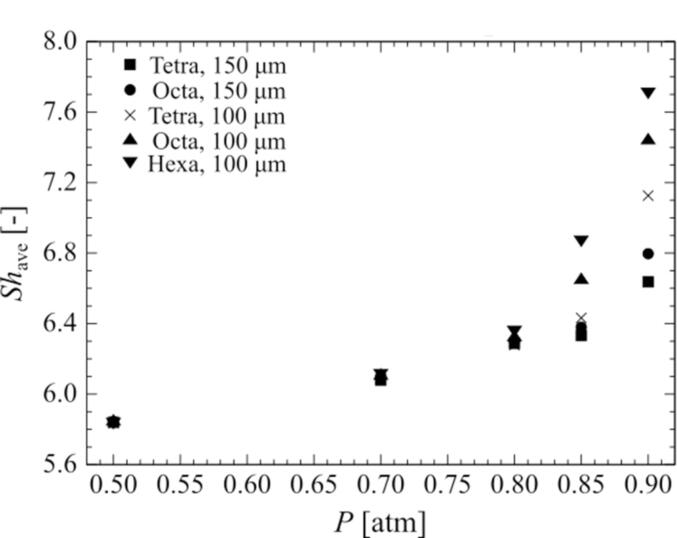
Relationship between the pressure amplitudes and the period-averaged Sherwood number of a surrounding bubble.

Fig. 10 shows the relationship between the cover ratio and (A) the mass transfer coefficient and (B) the mass flux of the surrounding bubble in an ultrasonic cycle. The time-averaged mass transfer coefficient and mass flux are defined as follows:

(28) $$k_{ave}=\frac{\int_{0}^{\frac{2\pi }{\omega }}k\left(t\right)dt}{\left(\frac{2\pi }{\omega }\right)}$$

(29) $$N_{ave}=\frac{\int_{0}^{\frac{2\pi }{\omega }}N\left(t\right)dt}{\left(\frac{2\pi }{\omega }\right)}$$

As shown in Fig. 10, these physical quantities increase with the cover ratio, and there is a positive linear relationship between the cover ratio, and time-averaged mass transfer coefficient, and mass flux.

Fig. 11 shows the relationship between the cover ratio and the *Sh* number of a surrounding bubble in an ultrasonic cycle. The time-averaged *Sh* number is defined as follows:

(30) $$Sh_{ave}=\frac{\int_{0}^{\frac{2\pi }{\omega }}Sh\left(t\right)dt}{\left(\frac{2\pi }{\omega }\right)}$$

As shown in Fig. 11, the time-averaged *Sh* number increases with an increase in the cover ratio, and there is also a positive linear relationship between the cover ratio and the time-averaged *Sh* number.

The mass transfer characteristics are proportional to the cover ratio because of the movement distance of the surrounding bubbles. Fig. 12 shows the relationship between the cover ratio and the movement distance of the surrounding bubble. The bubbles movement distance is proportional to the cover ratio. Therefore, as the cover ratio increases, the secondary Bjerknes force, that is, the attractive force between the bubbles, increases, causing an increase in the movement distance of the surrounding bubbles. The denser the bubbles, the larger the movement of the surrounding bubbles, and the greater the mass transfer out of the bubbles.

Finally, we discuss the effect of pressure amplitude on mass transfer across the bubble interface. Fig. 13 shows the relationship between the pressure amplitude and the time-averaged (A) mass transfer coefficient and (B) the mass flux of the surrounding bubble, and Fig. 14 shows the relationship between the pressure amplitude and the time-averaged *Sh* number of the surrounding bubble. For all arrangements of the surrounding bubbles, the physical quantities increase with increasing pressure amplitude. Moreover, the denser the bubbles, that is, the higher the cover ratio becomes, the larger the rate of increase in the physical quantities becomes. From 0.70 atm. to 0.90 atm., the increase in the physical quantities becomes very large with increasing pressure amplitude. In particular, for relatively higher cover ratios, they increase exponentially. This is due to the change in the bubble oscillation from linear to nonlinear, which enlarges the secondary Bjerknes force and increases the displacement of the surrounding bubbles. It should be noted that the mass transfer around the bubbles can be arranged by the cover ratio at different pressure amplitudes other than 0.90 atm., and similar results were obtained at other pressure amplitudes.

In this study, we investigated the effect of surrounding bubbles and their arrangement on the mass transfer at a frequency of 20 kHz. The chemical effect of the ultrasonic irradiation increased with increasing ultrasonic frequency. Therefore, further investigation of mass transfer at higher ultrasonic frequencies is necessary. As investigated in our previous study [51], a higher ultrasonic frequency increases the nonlinear bubble oscillations at a low-pressure amplitude and enhances the mass transfer toward the bubble outside because the concentration in the bubble becomes much higher in the case of a higher frequency at the compressed moment. Therefore, the effect of the surrounding bubbles on the mass transfer increases at higher ultrasonic frequencies. However, further studies are required to elucidate these phenomena at higher frequencies.

## 4 Conclusion

In this study, we numerically investigated the mass transfer of the chemical species around the acoustic cavitation bubbles in a multi-bubble environment. The main findings of this study are summarized as follows:

• At low pressure amplitudes, the positions of the bubbles do not change; therefore, the mass transfer of the chemical species in the bubble is small.
• At a high pressure amplitude, the secondary Bjerknes force becomes significant, and in addition, the asymmetric flow around the surrounding bubbles is generated, which causes the liquid jet to move toward the central bubble, causing the surrounding bubbles to move toward the central bubble.
• The chemical species are transferred to the outside of the bubbles especially during the compression period. As the secondary Bjerknes force becomes significant, the movement distance of the surrounding bubbles increases, increasing the amount of chemical species that are transferred to the outside of the bubbles.
• At a high pressure amplitude, there is a positive linear relationship between the cover ratio and the mass transfer characteristics to the bubble outside.
• Mass transfer to the outside bubble is enhanced with increasing pressure amplitude, and it is exponentially enhanced with increasing pressure amplitude at relatively higher cover ratios.

## CRediT authorship contribution statement

**Kanji D. Hattori:** Writing – original draft, Visualization, Validation, Investigation, Formal analysis, Data curation. **Takuya Yamamoto:** Writing – review & editing, Validation, Supervision, Software, Project administration, Methodology, Funding acquisition, Conceptualization.

## Declaration of competing interest

The authors declare that they have no known competing financial interests or personal relationships that could have appeared to influence the work reported in this paper.
